# Coordinated alpha and gamma control of muscles and spindles in movement and posture

**DOI:** 10.3389/fncom.2015.00122

**Published:** 2015-10-09

**Authors:** Si Li, Cheng Zhuang, Manzhao Hao, Xin He, Juan C. Marquez, Chuanxin M. Niu, Ning Lan

**Affiliations:** ^1^School of Biomedical Engineering, Med-X Research Institute, Shanghai Jiao Tong UniversityShanghai, China; ^2^School of Technology and Health, Royal Institute of TechnologyStockholm, Sweden; ^3^Department of Rehabilitation, Ruijin Hospital of School of Medicine, Shanghai Jiao Tong UniversityShanghai, China; ^4^Division of Biokinesiology and Physical Therapy, University of Southern CaliforniaLos Angeles, CA, USA

**Keywords:** α-γ motor system, propriospinal neurons, spinal circuits, muscle and spindle, computational modeling, simulation, movement and posture

## Abstract

Mounting evidence suggests that both α and γ motoneurons are active during movement and posture, but how does the central motor system coordinate the α-γ controls in these tasks remains sketchy due to lack of *in vivo* data. Here a computational model of α-γ control of muscles and spindles was used to investigate α-γ integration and coordination for movement and posture. The model comprised physiologically realistic spinal circuitry, muscles, proprioceptors, and skeletal biomechanics. In the model, we divided the cortical descending commands into static and dynamic sets, where static commands (α_*s*_ and γ_*s*_) were for posture maintenance and dynamic commands (α_*d*_ and γ_*d*_) were responsible for movement. We matched our model to human reaching movement data by straightforward adjustments of descending commands derived from either minimal-jerk trajectories or human EMGs. The matched movement showed smooth reach-to-hold trajectories qualitatively close to human behaviors, and the reproduced EMGs showed the classic tri-phasic patterns. In particular, the function of γ_*d*_ was to gate the α_*d*_ command at the propriospinal neurons (PN) such that antagonistic muscles can accelerate or decelerate the limb with proper timing. Independent control of joint position and stiffness could be achieved by adjusting static commands. Deefferentation in the model indicated that accurate static commands of α_*s*_ and γ_*s*_ are essential to achieve stable terminal posture precisely, and that the γ_*d*_ command is as important as the α_*d*_ command in controlling antagonistic muscles for desired movements. Deafferentation in the model showed that losing proprioceptive afferents mainly affected the terminal position of movement, similar to the abnormal behaviors observed in human and animals. Our results illustrated that tuning the simple forms of α-γ commands can reproduce a range of human reach-to-hold movements, and it is necessary to coordinate the set of α-γ descending commands for accurate and stable control of movement and posture.

## Introduction

The physiological system of human motor control is not only highly redundant (Bernstein, 1967; Martin et al., 2009), but also endowed with intricate dual α and γ sensorimotor control (Granit, 1975; Pierrot-Deseilligny and Burke, 2005; Lan and He, 2012; Prochazka and Ellaway, 2012). Our understanding about how movements are organized and how muscles are coordinated in performing different tasks remains incomplete due to lack of *in vivo* data during behaviors. One of the remaining issues in sensorimotor control is to account for the role of γ motor system in movement and posture. In spite of thorough elucidation of peripheral efferent and afferent innervations of the spindle organ (Matthews, 1964; Boyd, 1980; Hulliger, 1984; Prochazka, 1999), the importance of γ motor system in motor control is still not well understood.

A consistently observed phenomenon during both movement and posture control is α-γ co-activation (Vallbo, 1971; Taylor et al., 2004, 2006; Prochazka and Ellaway, 2012). Direct recording by Taylor et al. (2004, 2006) revealed a co-varying pattern of gamma-static and dynamic firings with joint angle during locomotion. There was plenty evidence of independent control of γ-motoneurons during movement (Prochazka et al., 1985; Dimitriou and Edin, 2008). The co-activation of γ motoneurons with α motor activity was generally viewed to compensate for the unloading effects of muscle contraction to spindle sensitivity. Using a realistic virtual arm (VA) model (He et al., 2013), Lan and He (2012) suggested a plausible function of γ_*s*_ fusimotor control to convey centrally encoded joint angle information to the periphery through regulating spindle sensitivity, so that the Ia signaling from spindle afferents is kept faithfully proportional to the joint angle during movement and muscle contraction. This not only explains γ co-activation with α activity, but also supports the hypothesis that the γ_*s*_ command reinforces the centrally planned kinematics of movement and posture by way of spinal circuits.

Neurophysiological studies have identified separate spinal pathways and circuits of sensorimotor system in details, where the α and γ commands interact with each other to produce sensory and motor outputs (Baldissera et al., 1981; Lemon et al., 2004; Pierrot-Deseilligny and Burke, 2005). In addition to the mono-synaptic cortico-motoneuronal pathway (Lawrence and Kuypers, 1968; Lemon, 1999; Quallo et al., 2012), disynaptic excitatory and inhibitory cortico-motoneuronal pathways via PNs in C3-C4 were found to exist in cats and in macaque monkeys, as well as in humans (Malmgren and Pierrot-Deseilligny, 1988; Gracies et al., 1994; Alstermark et al., 2007; Isa et al., 2007). The PN was shown to play an important role in reaching movement of upper limb (Alstermark et al., 1981; Alstermark and Isa, 2012). In a computational analysis to emulate involuntary oscillatory movements in human upper extremity (Hao et al., 2013), it is postulated that movement signals are transmitted via the disynaptic pathway of the PN network, where the γ-dynamic (γ_*d*_) command is integrated with the α-dynamic (α_*d*_) command to produce pre-motoneuronal outputs. The γ_*d*_ command encodes kinetic information of joint acceleration (or deceleration), and gates the α_*d*_ command of double frequency at the PN network to determine the timing of activation for a pair of flexor and extensor muscles during oscillatory movements.

These studies suggest that while the α motor system provides the main drive for muscles, the γ motor system executes a more subtle control for movement dynamics and the maintenance of posture. In this paper, we extend the α-γ model (Hao et al., 2013) to investigate the modular control of voluntary movement and posture, and to demonstrate coordination of a set of α-γ descending commands in the control of movement and posture. Human reach-and-hold movements and muscle EMG activities were recorded and analyzed to guide the specification of the central descending commands. The model behavior was matched to the data of human movement and posture in all subjects. A variety of modifications were introduced in model structures to assess the functional significance of α-γ coordination by “deafferenting” or “deefferenting” the model. Results of this study provided a quantitative evaluation of the relative importance that each proprioceptive afferent and descending α-γ command may have on movement and posture control. Agreement between model predictions and human movement data further corroborated the α-γ coordination as a rudimentary means of sensorimotor control. The results argue that coordinated γ control with α activation is essential for accurate and stable control of movement and posture. Preliminary analysis of this study appeared in a conference proceeding (Li et al., 2014).

## Materials and methods

### Corticospinal virtual arm (CS-VA) model

**Figure 1:**
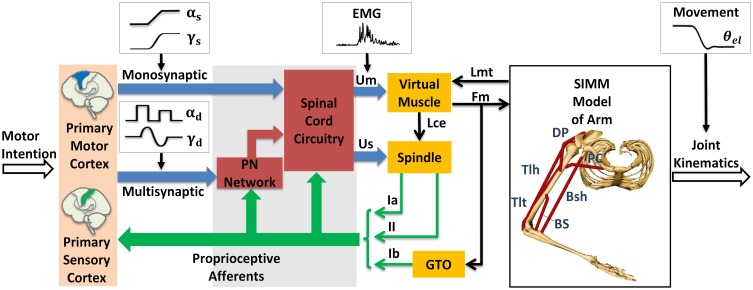
**Corticospinal virtual arm (CS-VA) model**. Descending α and γ commands from motor cortex were processed in PN network and spinal cord circuitry, the coordinated output of α and γ motor neurons are transmitted to virtual muscles and spindles, the virtual arm is then activated. PN represents propriospinal interneuron. The subscripts “s” and “d” in α and γ motor signals stand for static and dynamic commands, respectively. *U*_*m*_ is muscle input, which is equivalent to human EMG; *U*_*s*_ is spindle input; *L*_*mt*_ and *F*_*m*_ represent muscle tendon length and muscle force computed form virtual arm model; *L*_*ce*_ represents fascicle length; Ia and II are sensory feedback of primary and secondary afferents from muscle spindle; and Ib is the feedback of Golgi Tendon Organ (GTO). The virtual arm has two joints including shoulder and elbow in the horizontal plane, with two degrees of freedom, i.e., shoulder flexion/extension and elbow flexion/extension. Three pairs of antagonistic muscles are included in the model: shoulder flexor Pectoralis Major Clavicle (PC), extensor Deltoid Posterior (DP), elbow flexor Brachialis (BS), extensor Triceps Lateral (Tlt), and biarticular flexor Biceps Short Head (Bsh), extensor Triceps Long Head (Tlh). Simulated joint kinematics, especially elbow angle is compared to elbow angle (θ_*el*_) recorded in human experiment. Modified from Hao et al. (2013) with permission.

**Figure 2:**
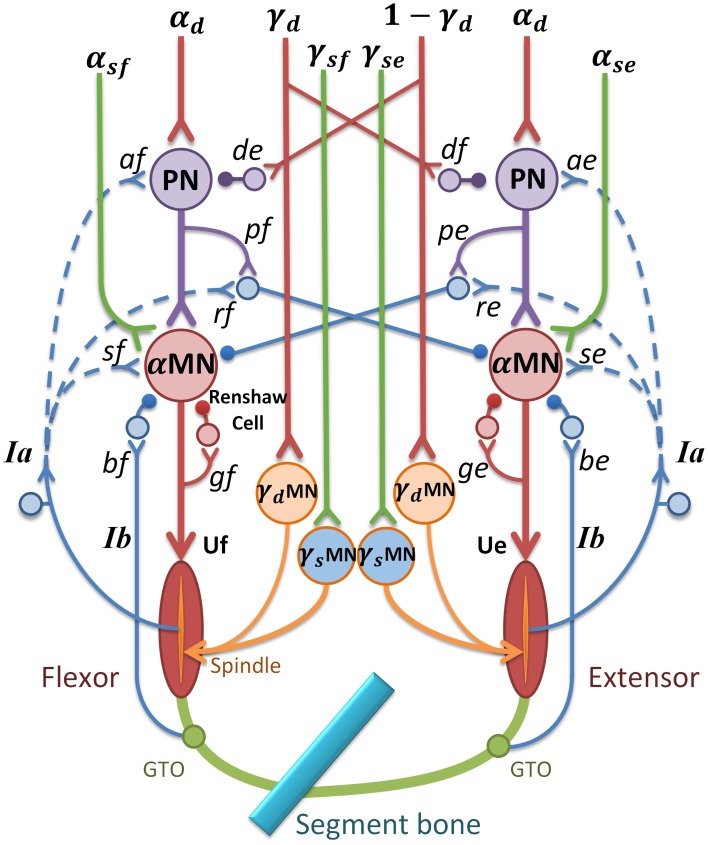
**α-γ control model in spinal cord circuitry for a pair of antagonistic muscles**. α and γ commands are coordinated by PN network and reflex circuitry within spinal cord. The subscripts “e” and “f” stand for extensor and flexor; MN represents motor neuron pool; U represents muscle input; *d*_*e*_ and *d*_*f*_ are gains of γ_*d*_ inhibition on PN(γ-PN); *p*_*e*_ and *p*_*f*_ are gains of PN to reciprocal inhibition; *g*_*e*_ and g_*f*_ are gains of recurrent inhibition from Renshaw Cell (RC); *b*_*e*_ and *b*_*f*_ represent inhibition gains on α motor neurons from Ib afferents (Ib(−)); *s*_*e*_ and *s*_*f*_ are stretch reflex gains from Ia afferents ((Ia(+)); *r*_*e*_ and *r*_*f*_ are reciprocal inhibition gains on α motor neurons (Ia(−)); *a*_*e*_ and *a*_*f*_ are Ia afferent gains on PN(Ia-PN). Excitatory synapse at neurons is indicated by a “y” termination, and inhibitory synapse is indicated by a filled dot termination. The function of the feedback from Ia afferents in dashed lines are discussed later.

The module of posture and movement control based on α-γ dual control system was implemented in the computational CS-VA model (Figure 1). This model is based on realistic physiological studies (Cheng et al., 2000; Mileusnic and Loeb, 2006; Mileusnic et al., 2006; Taylor et al., 2006; Alstermark et al., 2007; Isa et al., 2007) and has been validated to capture the realistic neuromechanical properties of human upper limb in previous work (Song et al., 2008a,b; Lan and He, 2012; He et al., 2013). It consists of four parts, the primary motor and sensory cortex, PN network, spinal cord circuitry and virtual arm (VA) model. The spinal cord network with PNs innervating a pair of antagonistic muscles (Figure 2) has been validated in the mechanism study of Parkinsonian tremor (Hao et al., 2013). The VA has two joints including shoulder and elbow in the horizontal plane, two degrees of freedom, namely shoulder flexion/extension and elbow flexion/extension. The six dominating muscles includes two pairs of monoarticular muscles, shoulder flexor Pectoralis Major Clavicle (PC), extensor Deltoid Posterior (DP), elbow flexor Brachialis (BS), extensor Triceps Lateral (Tlt) and one pair of biarticular muscles, flexor Biceps Short Head (Bsh), extensor Triceps Long Head (Tlh). Muscle spindle and Golgi Tendon Organ (GTO) are implemented within the VA to provide proprioceptive feedback. Three parts of the CS-VA model, including PN network, spinal cord circuitry and VA, have been integrated in SIMULINK/MATLAB platform. In this study, bi-articular muscles of Bsh and Tlh were mainly used to realized movement and posture.

Within the CS-VA model, central motor commands from motor cortex are transmitted to spinal α and γ motoneurons (αMN, γMN) in two pathways, mono-synaptic pathway carrying static α and γ motor commands to corresponding motoneurons for posture control, and multi-synaptic pathway transmitting dynamic α and γ motor commands to MNs via PN network for movement control. Muscles and spindles are activated by α and γ motor signals respectively after the regulation of PN and reflex network within spinal cord. The muscular dynamics computed by SIMM model of arm give a real-time muscle tendon length to virtual muscle, at the same time, the dynamics are reflected by Ia and II afferents from muscle spindle, and Ib afferent from GTO. Feedback from proprioceptive afferents participates in signal processing within spinal cord network and cortex simultaneously. In this framework, central inputs of static and dynamic α and γ motor commands are specialized in Section Specification of Central Descending Commands, the input of virtual muscle (*U*_*m*_) is equivalent to recorded EMG, and the output joint kinematics gives a behavioral indicator to compare with elbow angle (θ_*el*_) in human experiment.

PNs in C3-C4 receive wide descending control from cortico-, rubro-, reticulo and tectospinal tracts, and project inhibition or excitation to downstream αMN (Alstermark et al., 2007), at the same time receive feedback from Ia and cutaneous afferents (Alstermark et al., 1984a,b,c). Based on neurophysiology structures of PN network, αMN, γMN, and the spinal reflexes, a model of spinal circuitry was implemented within the CS-VA model as is shown in Figure 2 (Hao et al., 2013; Li et al., 2014). The central descending commands in the model were passed down to αMN and γMN through mono-synaptic and multi-synaptic PN pathways, respectively (Isa et al., 2007). There was experimental evidence indicating that PNs mediate motor commands for reaching movement (Alstermark et al., 2007; Alstermark and Isa, 2012). Computational analysis (Lan and He, 2012) suggested that γ_*s*_ conveys kinematic information of joint angle. Therefore, in this model, we assume that the descending commands can be divided into static set (α_*s*_ and γ_*s*_) for posture and dynamic set (α_*d*_ and γ_*d*_) for movement. This division is consistent to the evidence of individual posture and movement modules in central motor control system (Kurtzer et al., 2005).

As illustrated in Figure 2, α and γ commands control muscles and spindles through a set of spinal circuits. α_*d*_ is integrated at the PN with γ_*d*_ in a pattern of mirror inhibition (γ_*d*_ or *1*-γ_*d*_). The recording from dynamic γMN in Taylor et al. (2006) revealed a reciprocal change of γ_*d*_ with joint angle. The PN also receives excitation from autogenic Ia afferent (Ia-PN) (Alstermark et al., 1984b; Malmgren and Pierrot-Deseilligny, 1988). After the PN, α_*s*_ and α_*d*_ commands converge at αMN, and its output is further regulated by reciprocal inhibition (Ia(−)) from antagonistic muscle, recurrent inhibition of Renshaw Cell (RC), autogenic stretch reflexes from Ia (Ia(+)), and Ib afferents (Ib(−)) (Eccles et al., 1957, 1960; Windhorst, 2007). The output of PN (*Y*_*PN*_) and αMN (*Y*_α__*MN*_) are given in Equation (1), in which subscript “e” and “f” represent extensor and flexor,

(1a) $$Y_{PN}{}_{e}=\alpha_{d}-d_{f}*\gamma_{d}+a_{e}*\upsilon_{e}\prime$$

(1b) $$Y_{PN}{}_{f}=\alpha_{d}-d_{e}*(1-\gamma_{d})+a_{f}*\upsilon_{f}\prime$$

(1c) $$N_{e}\left(t\right)=\alpha_{s}{}_{e}+Y_{PN}{}_{e}$$

(1d) $$N_{f}\left(t\right)=\alpha_{s}{}_{f}+Y_{PN}{}_{f}$$

(1e) $$\frac{dC_{e}(t)}{dt}=-\frac{1}{\tau_{N_{e}}}C_{e}(t)+\frac{1}{\tau_{N_{e}}}N_{e}\left(t\right)0\leq N_{e}\left(t\right)\leq 1$$

(1f) $$\frac{dC_{f}(t)}{dt}=-\frac{1}{\tau_{N_{f}}}C_{f}(t)+\frac{1}{\tau_{N_{f}}}N_{f}\left(t\right)0\leq N_{f}\left(t\right)\leq 1$$

(1g) $$C_{e}\prime (t)=C_{e}(t)\times \sigma (t)$$

(1h) $$C_{f}\prime (t)=C_{f}(t)\times \sigma (t)$$

(1i) $$Y_{\alpha MN}{}_{e}=\frac{C_{e}\prime (t)}{1+g_{e}C_{e}\prime (t)}\left[1+s_{e}\upsilon_{e}\prime -r_{e}\upsilon_{f}\prime -b_{e}\varphi_{e}\prime \right]$$

(1j) $$Y_{\alpha MN}{}_{f}=\frac{C_{f}\prime (t)}{1+g_{f}C_{f}\prime (t)}\left[1+s_{f}\upsilon_{f}\prime -r_{f}\upsilon_{e}\prime -b_{f}\varphi_{f}\prime \right]$$

*N*(*t*) is the sum of descending commands to αMN, *C(t)* represents background activation of αMN pools, τ is the time constant of excitation with the value 0.029 (sec), σ(*t*) represents the signal dependent noise, which is a Gaussian distributed random signal (Fuglevand et al., 1993; Jones et al., 2002). *C*(*t*) was multiplied by σ(*t*) to yield the noise corrupted background activations (*C*′(*t*)) in Equations (1g) and (1h). The output of αMN (Equations 1i and 1j) is the sum of all excitatory and inhibition inputs; here, the values of υ′ and φ′ are proportional to Ia and Ib afferent discharge frequencies, respectively; and *g, s, r, b* are reflex gains of RC, Ia(+), Ia(−), Ib(−). The values of spinal reflex gains are listed in Table 1.

**Table 1:** **Reflex gains of spinal circuitry in the CS-VA model**.

**Muscles**	**PN and Reflex gains**						
	***a***	***d***	***p***	***r***	***s***	***g***	***b***
PC	0.1	1	0	0.1	0.2	0.2	0.1
DP	0.1	1	0	0.1	0.2	0.2	0.1
Bsh	0.1	1	0	0.1	0.2	0.2	0.1
Tlh	0.1	1	0	0.1	0.2	0.2	0.1
BS	0.1	1	0	0.1	0.2	0.2	0.1
Tlt	0.1	1	0	0.1	0.2	0.2	0.1

*a, Ia afferent gain on PN (Ia-PN); d, γ dynamic inhibition gain on PNs(γ-PN); p, gain of PN to reciprocal inhibition; r, Ia reciprocal inhibition gain (Ia(−)); s, stretch reflex gain (Ia(+)); g, recurrent inhibition gain from Renshaw Cell (RC); b, Ib gain of Golgi tendon organ (Ib(−))*.

### Human reach and hold experiment

#### Subjects and experiments

Seven healthy adults participated in this elbow joint's reach and hold experiment. The human subject study was approved by the Internal Review Board (IRB) of University of Southern California (USC). All of them obtained a brief explanation of this study before the experiment, and signed the informed consent.

**Figure 3:**
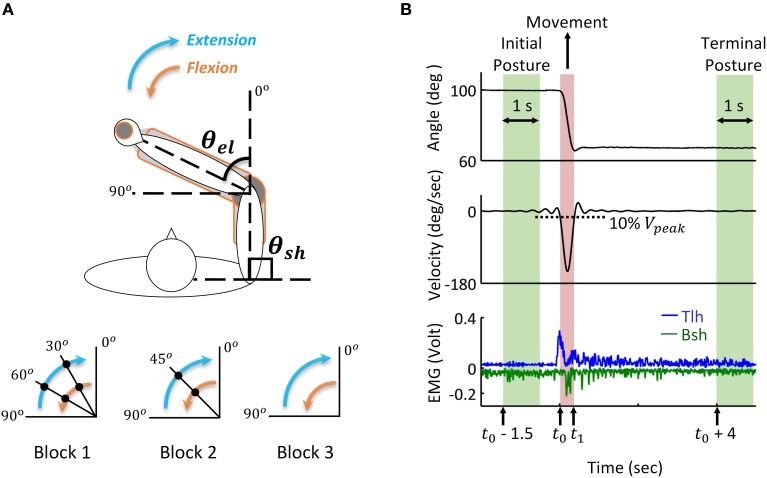
**Human reach and hold experiments, and analysis of kinematic and EMG data during posture and movement. (A)** Experimental set up: the subject is seated at the table to make fast reaching movement of elbow joint in the horizontal plane. Subjects' arm and hand were secured on a manipulandum to keep the upper arm stable and ensure smooth extension and flexion of the forearm. Elbow angle (θ_*el*_) is defined as the included angle between forearm and the extending line of upper arm. Movements with a range of 30, 45, and 90 degrees are performed in Block 1, 2, and 3, respectively. During extension, the elbow angle (θ_*el*_) changes from initial posture of 90° to terminal posture of 0°, the filled dot indicates the holding posture between reaching movements. The reversal flexion from 0° to 90° has the same movement range and holding postures. The shoulder angle (θ_*sh*_) kept at 90° during all experiments. **(B)** Analysis of kinematic and EMG data during posture and movement. During movement, the elbow moves from initial posture to terminal posture, and the movement onset (*t*_0_) and offset (*t*_1_) are defined as the time at which the velocity change (increases or decreases) is 10% of the peak velocity (*V*_*peak*_). Initial posture period starts at time (*t*_0_ – 1.5) and terminal posture is defined from time (*t*_0_ + *4*) after the angle stabilizes. The time window is 1 s. The tri-phasic EMG pattern of extensor Triceps long head (Tlh) and flexor Biceps short head (Bsh with inverse value) is shown in the bottom (low passed at a cut off frequency of 50 Hz with digital Butterworth filter).

During the experiment, the subject sat comfortably with the upper arm maintained perpendicular to the trunk in the horizontal plane (Figure 3A), the hand and forearm were secured on the manipulation to make single joint movements around the elbow. All subjects initially held their elbow at 90°, and performed successive reach and hold movements triggered by the visual cue of LED light, following three blocks. In Block 1, the elbow was extended with the range of 30 degrees, and after three successive extensions, the elbow stopped at 0°, the reversal flexion with the same movement range and holding posture was performed after a 5 s holding period at 0°. In Block 2, the range of extension and flexion was 45°, thus one holding period at the mid-posture of 45° was performed, and in Block 3 the elbow had direct extension and flexion movement between 90° and 0°. During the experiments, the subjects moved as fast as them could between postures, and a 5 s holding was required at each posture. Each block was repeated for five trials, and between blocks, the subjects had more than 10 min to rest. During the movement, the elbow angle was recorded and EMGs of biceps short head (Bsh) and triceps long head (Tlh) were collected using bi-polar surface electrodes, the EMG signals were pre-amplified at the gain of 1000, band-pass filtered with cut-off frequencies at 5 Hz and 1000 Hz, and then sampled at 2000 Hz for post-processing.

#### Analysis of kinematics and EMG data

Recorded joint trajectory and EMG were analyzed to provide guidance for simulation. Since Subject 3 couldn't follow the experimental protocol, the data was excluded from result analysis. The joint angles were low-pass filtered with a cut-off frequency of 10 Hz to remove high-frequency noise, and differentiated to obtain velocity. The collected EMG of biceps and triceps were band-pass filtered with a cut-off frequency between 20 and 500 Hz to remove motion artifacts and high-frequency noise, rectified, and then low passed at the cut-off frequency 50 Hz for further analysis. As shown in Figure 3, the time for movement onset (*t*_0_) and offset (*t*_1_) was defined as the time where velocity increased and decreased to 10% of peak velocity (*V*_*peak*_) respectively (Atkeson and Hollerbach, 1985). The static angles and EMG during initial posture were then defined as the averaged value from time (*t*_0_−1.5) (sec) to time (*t*_0_−0.5) (sec) before movement onset. Terminal posture phase started from time (*t*_1_ + 4) (sec), and steady state posture angle was obtained in a window of 1 (sec). The onset of muscle firing was calculated as the time at which EMG amplitude exceeded static EMG for three times of standard deviation (3SD) of it, and maintained higher than it for at least 25 ms (Hodges and Bui, 1996; Takatoku and Fujiwara, 2010), the offset of muscle firing was calculated as the time EMG decreased lower than 3 SD below static EMG. According to this threshold criterion, the duration of muscle firing was obtained, and a low passed EMG at the cut off frequency of 6 Hz (Steele, 1994) was used to detect the amplitude of EMG. Off-line Digital Butterworth filters were used in this study with forward and reverse passes to avoid phase shift. The static and dynamic features of movements and EMGs were used to tune parameters of descending commands for corresponding posture and movement.

### Specification of central descending commands

To fit the behaviors of the model in Section Corticospinal Virtual Arm (CS-VA) Model to the experimental data in Section Human Reach and Hold Experiment, the parameters of the model are fixed throughout the simulation. Only parameters of central descending commands are adjusted to capture the realistic feature of human movements and postures, as described in the following sub-sections.

#### Determining static command set (α_*s*_, γ_*s*_) for posture

Posture was realized by adjusting static α and γ commands in this model. Earlier results (Lestienne et al., 1981) indicated that terminal posture could be coded as the ratio of activation levels between antagonistic muscles of a joint, thus α_*s*_ of Bsh and Tlh in the simulation was set according to the relationship between ratio of static EMG (Bsh/Tlh) (RS) and static elbow angle (θ_*el*_) of human experiment (Equation 2). The initial and terminal α_*s*_ of Bsh and Tlh were then decided by corresponding RS at angle θ_*el*_ from the experiment. The value of α_*s*_ was adjusted in the model to keep a low activation on muscles during postures. The transition of α_*s*_ between initial and terminal postures was assumed as a ramped changing pattern. To ensure a single joint movement in the simulation, the shoulder was fixed through adding a constant high co-activation level on α_*s*_ of PC and DP.

(2) $$\frac{\alpha_{s}\left(\theta_{el,}Bsh\right)}{\alpha_{s}\left(\theta_{el},Tlh\right)}=RS_{\left(\theta_{el}\right)}$$

γ_*s*_ is related to centrally planned kinematics. In this study, we adopt an experimentally approved criterion for the central planned angle trajectory, the minimal-jerk criterion (Hogan, 1984; Flash and Hogan, 1985). The objective function is given in Equation (3a), and the minimal-jerk trajectory (MJT) is obtained by integration from *t*_0_ and *t*_1_, which are times of movement onset and end. The outcome is a smooth angle trajectory in Equation (3b), as follows.

(3a) $$Z=\frac{1}{2}\int_{t_{0}}^{t_{1}}{\left(\frac{d^{3}\theta }{d^{3}t}\right)}^{2}dt$$

(3b) $$\theta_{el}(t)=\sum_{i\;=\;0}^{5}a_{i}t^{i}$$

The γ_*s*_ input for muscle spindles was calculated from the MJT during posture and movement, according to the quadratic relationship between γ_*s*_ and joint angle obtained in Lan and He (2012). The γ_*s*_ inputs to Bsh and Tlh were determined in Equations (4a) and (4b) in the following:

(4a) $$\gamma_{s}\left(Bsh\right)=2e-5{\left(\theta_{sh}+\theta_{el}\right)}^{2}-\;0.0008\left(\theta_{sh}+\theta_{el}\right)+\;0.3698$$

(4b) $$\gamma_{s}\left(Tlh\right)=8e-6{\left(\theta_{sh}+\theta_{el}\right)}^{2}-\;0.0044(\theta_{sh}+\theta_{el})\;+\;0.9292$$

where the shoulder angle (θ_*sh*_) was fixed, and the elbow angle trajectory (θ_*el*_) was the MJT from Equation (3b). The unit of joint angles in Equation (4) is degree.

#### Determining dynamic command set (α_*d*_, γ_*d*_) for movement

According Taylor's recording (Taylor et al., 2000, 2004, 2006), γ_*d*_ efferent had a constant baseline of firing rate during both posture and movement, and its firing rate sensitively changed with onset of muscle lengthening, thus presented a leading phase before movement. In the previous analysis of involuntary oscillatory movements (Hao et al., 2013), it was hypothesis that γ_*d*_ represented joint acceleration, and was used to steer activations of antagonistic muscles for joint acceleration and deceleration. This reproduced all features of involuntary oscillatory movements such as Parkinsionian tremor. Thus in the present study, we also adopted this hypothesis, and determined the γ_*d*_ command as the acceleration of the MJT as follows:

(5) $$\gamma_{d}=0.5+\;k\frac{d^{2}\theta_{el}(t)}{d^{2}t}$$

In which θ_*el*_(*t*) was adopted from Equation (3b). The constant bias was set at 0.5 (normalized) to give a balanced (or mirror) inhibition on antagonistic PNs (see Figure 2). Parameter *k* was chosen so that the value of γ_*d*_ was within 0 and 1.

Movement was performed through integrating dynamic α and γ commands at the PN network. It has been proposed that, the motor system modulated the pulse amplitude and duration of muscle activations to produce movement with different velocities and ranges (Corcos et al., 1989; Gottlieb et al., 1989), and the pulse strategy has successfully generated scaled movements (Lan, 1997; Lan et al., 2005). Thus, a pair of pulses was utilized on α_*d*_ to act as agonistic acceleration (AG1) and antagonistic deceleration (ANT) phase respectively. The pulse waveform was given in Equations (6a) and (6b):

(6a) $$\alpha_{d}=\{\begin{array}{l} amp_{AG1},t\in \left(t_{AG1},t_{AG1}+pw_{AG1}\right)\\ amp_{ANT},t\in \left(t_{ANT},t_{ANT}+pw_{ANT}\right)\\ 0,t\in \left(0,t_{AG1}\right)\cup (t_{AG1}+pw_{AG1},t_{ANT})\\ \;\cup \left(t_{ANT}+pw_{ANT},t_{sim}\right) \end{array}$$

(6b) $$\frac{amp_{AG1}}{amp_{ANT}}=RP$$

where *amp*_*AG*1_ and *amp*_*ANT*_ were pulse amplitudes of AG1 and ANT (for extension movement, AG1 is on Tlh and ANT is on Bsh respectively), and their values were based on the ratio of peak EMG (ANT/AG1) (RP) obtained in human movement. *pw*_*AG*1_ and *pw*_*ANT*_ were the pulse widths for AG1 and ANT, which were set as the durations of experimental EMGs according to the 3SD threshold criterion in Section Analysis of Kinematics and EMG Data. *t*_*AG*1_ and*t*_*ANT*_ were the starting times for AG1 phase and ANT phase, *t*_*sim*_ was the terminal time of simulation. Therefore, by adjusting the amplitude and width of the bi-phasic pulses, movements with different ranges and velocities could be achieved by the model.

#### Determining the stabilization pulse

A third pulse with a ramped decreasing form was necessary to stabilize the joint after movement. It was implemented in α_*s*_ of agonist muscle (second agonistic muscle burst, AG2), since tri-phasic patterned EMG is found a common feature in fast reaching movement (Ghez and Martin, 1982; Flanders et al., 1994; Berardelli et al., 1996), and AG2 has been widely accepted as a phase to help stabilize the elbow at terminal posture after movement (Hannaford and Stark, 1985; Takatoku and Fujiwara, 2010). The amplitude and duration of AG2 were adjusted in the simulation to stabilize the joint after movement.

### Simulation with intact feedforward and feedback controls

Normal condition with intact feedforward and feedback control was simulated to match typical trials in human experiment. For each subject, different postures were simulated and compared to the experimental relationship between RS and steady state angle. Stiffness control by varying co-activation level of antagonist muscles at different postures was demonstrated. To determine joint stiffness, a force perturbation was added directly on single joint muscle BS, and the stiffness was calculated as the ratio of changes in elbow torque with respect to joint angle (He et al., 2013). For each subject, the extension movement from 60° to 30° (Movement 1) was specifically simulated and matched to experimental data. More simulations were performed to fit experimental movements of larger range and in opposite direction for Subject 1, Simulation of 11 s was usually performed. After the system had converged at a steady initial state, a random, signal dependent noise (Jones et al., 2002) was added on *U*_*m*_, the elbow was driven at time 6 (sec). Reflex gains used in this study were set within the range that kept the system stable (Table 1) (He et al., 2013). All α and γ commands used in simulation were normalized to values between 0 and 1. Nominal parameter values of spinal circuits were adopted from Hao et al. (2013), and were listed in Table 1.

### Simulation with abnormal feedforward and feedback controls

In this study, we examined the effects of altering model structure on movement and posture by deafferenting and deefferenting the model. Deafferented conditions were modeled by assigning the related gains of Ia, and Ib to zero. Deefferented conditions of the model were obtained by setting one of the descending commands to zero at a time, while keeping others intact. The effect of abnormal ratio (Bsh/Tlh) of α_*s*_ on terminal postures was also examined by assigning static ratio out of normal ranges of experiments. Note that γ_*d*_ was always maintained a bias of 0.5 in the analysis of deefferentation study. The abnormal conditions were applied to the model 0.1 (sec) before movement initiation in simulations.

The difference between experimental and simulated movements was used to quantify the effects of abnormal control of movement and posture. Angular errors for movements and terminal steady state posture were evaluated as in Equations (7a) and (7b):

(7a) $$Error_{P}=\frac{1}{W}\sum_{j=1}^{W}\left(\theta_{el(P_{sim}(j))}-\theta_{el(P_{exp}(j))}\right)$$

(7b) $$Error_{M}=\frac{1}{V}\sum_{j=1}^{V}\left(\theta_{el(M_{sim}(j))}-\theta_{el(M_{exp}(j))}\right)$$

where *Error*_*P*_ and *Error*_*M*_ gave the averaged differences in terminal steady state posture and movement between simulated and experiment respectively (Figure 3), W and V were the number of resampled data points during terminal steady state posture and movement phases.

## Results

### Features in kinematics and EMGs of human posture and movement

All subjects presented a common pattern in movement and posture at the elbow joint. The joint angle was stabilized after a slight overshoot, and the velocity displayed a bell-shaped profile. Tri-phasic EMG was observed in fast movements, with AG1 firing at accelerating phase, ANT at deceleration phase. The AG2 of agonist muscle appeared during movement offset and lasted until the joint was stabilized at terminal posture. When the elbow was stabilized after movement, the EMG of Tlh and Bsh were maintained at different steady state levels, which varied with elbow angle.

**Figure 4:**
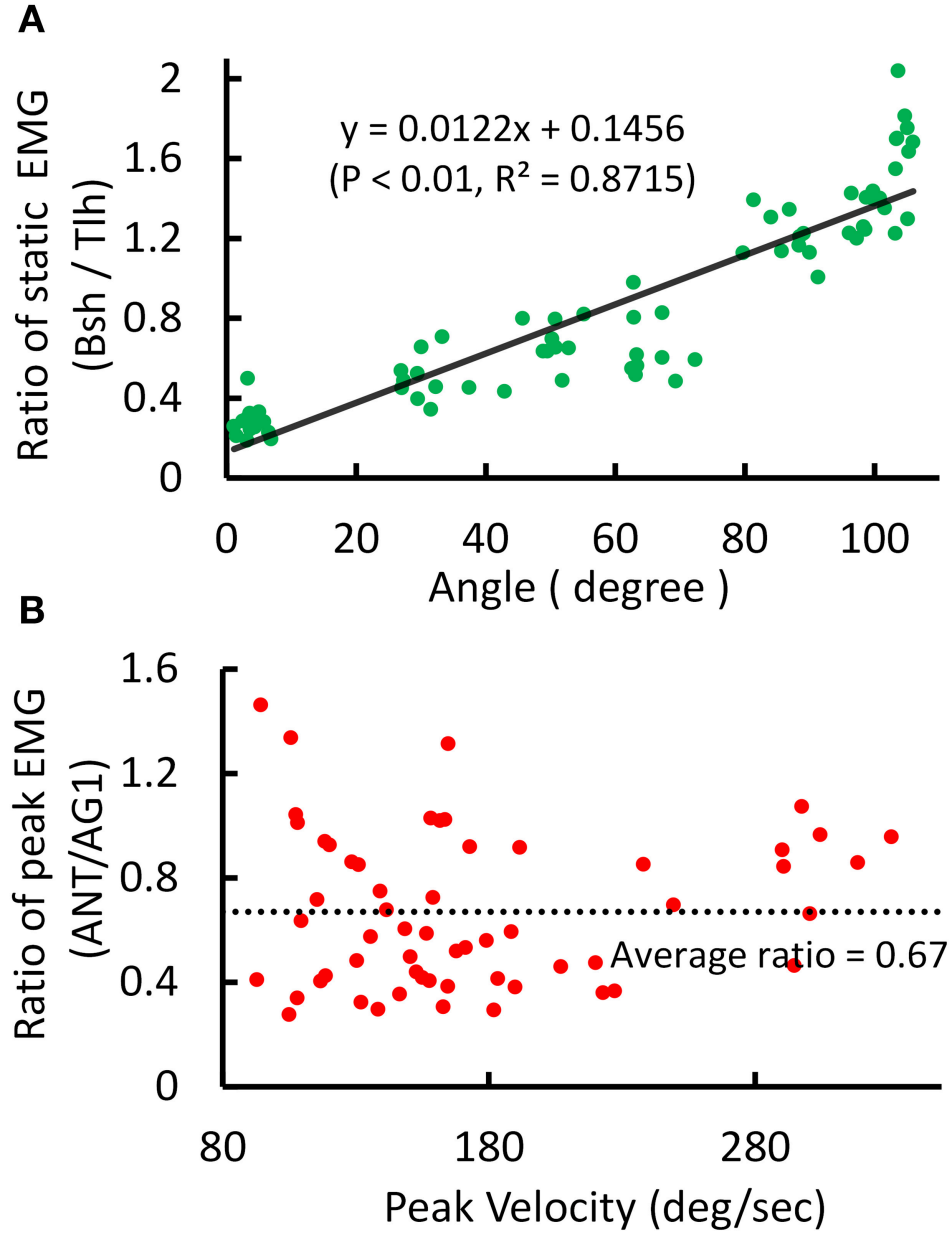
**Experimental kinematic and EMG during posture and movement of Subject 1. (A)** Relationship between postural EMG ratio of Biceps and Triceps and elbow angle, and a linear regression is fitted with *P* < 0.01, *R*^2^ = 0.8715. **(B)** Relationship between the ratio of peak EMG (ANT/AG1, Bsh/Tlh for extension, Tlh/Bsh for flexion) and peak velocity for fast reaching. The linear regression is rejected (intercept *P* > 0.05).

An example of the relations between EMG ratio and joint angle during posture and movement was presented in Figure 4. The EMG of Tlh and Bsh were found to vary reciprocally with the elbow angle from 0° to 110°. The ratio of static EMG (Bsh/Tlh) (RS) was well fitted against the elbow angle in a linear manner (Figure 4A, *P* < 0.01). During movement although the peak velocity increased with movement range and speed, The relationship between peak velocity and ratio of peak EMG (ANT/AG1) (RP) was almost flat (*P* > 0.05), as shown in Figure 4B, and the ratio of peak EMG had an average value of 0.67 (±0.29).

These features were present in all subjects. A summary of experimental relation between the ratio of static EMGs and elbow angle for all 6 subjects was given in Table 2. This indicates that the central module for posture control purposefully varies the static levels of antagonistic muscle activation in such particular linear manner to compensate for the change in moment arms of muscles at different joint angles. This experimental linear relationship is sufficient to guide the specification of α_*s*_ descending commands in the model for posture maintenance.

**Table 2:** **Linear relationship between ratio of static EMG (RS) and elbow angle (θ_*el*_) (Experiment (Exp.) vs. Simulation (Sim.))**.

**Subject**	**Exp./Sim**.	***RS* = *K* × θ_*el*_+ *M***		
		***K***	***M***	***R^2^***
S1	Exp.	0.0122	0.1456	0.8715
	Sim.	0.0118	0.2328	0.9347
S2	Exp.	0.0072	0.5422	0.8099
	Sim.	0.0083	0.4729	0.6169
S4	Exp.	0.0165	0.3871	0.6164
	Sim.	0.0179	0.3076	0.8885
S5	Exp.	0.0093	0.4967	0.6278
	Sim.	0.0064	0.5113	0.7044
S6	Exp.	0.0191	0.4387	0.6848
	Sim.	0.0132	0.6275	0.7597
S7	Exp.	0.0095	0.3848	0.8560
	Sim.	0.0123	0.3177	0.9538
Average	Exp.	0.0119	0.3992	0.5306
	Sim.	0.0112	0.4075	0.3954

### Simulated posture control

Posture control was then simulated by tuning α_*s*_ inputs to Bsh and Tlh for each subject, and by setting γ_*s*_ according to the static angle θ_*el*_. Using the linear relation to guide the specification of α_*s*_ commands for Bsh and Tlh muscles in the model, a comparable relationship was obtained in simulated postures for all subjects. The results in Table 2 showed that the simulated linear equations for each subject matched to the experimental relationship closely (*P* < 0.05). Overall an average linear relationship existed in experiment and simulated groups for all six subjects (*P* < 0.05), and they had a similar slope and intercept. This indicates that posture control can be achieved by tuning α_*s*_, as well as setting γ_*s*_ inputs to relevant antagonistic muscles.

**Figure 5:**
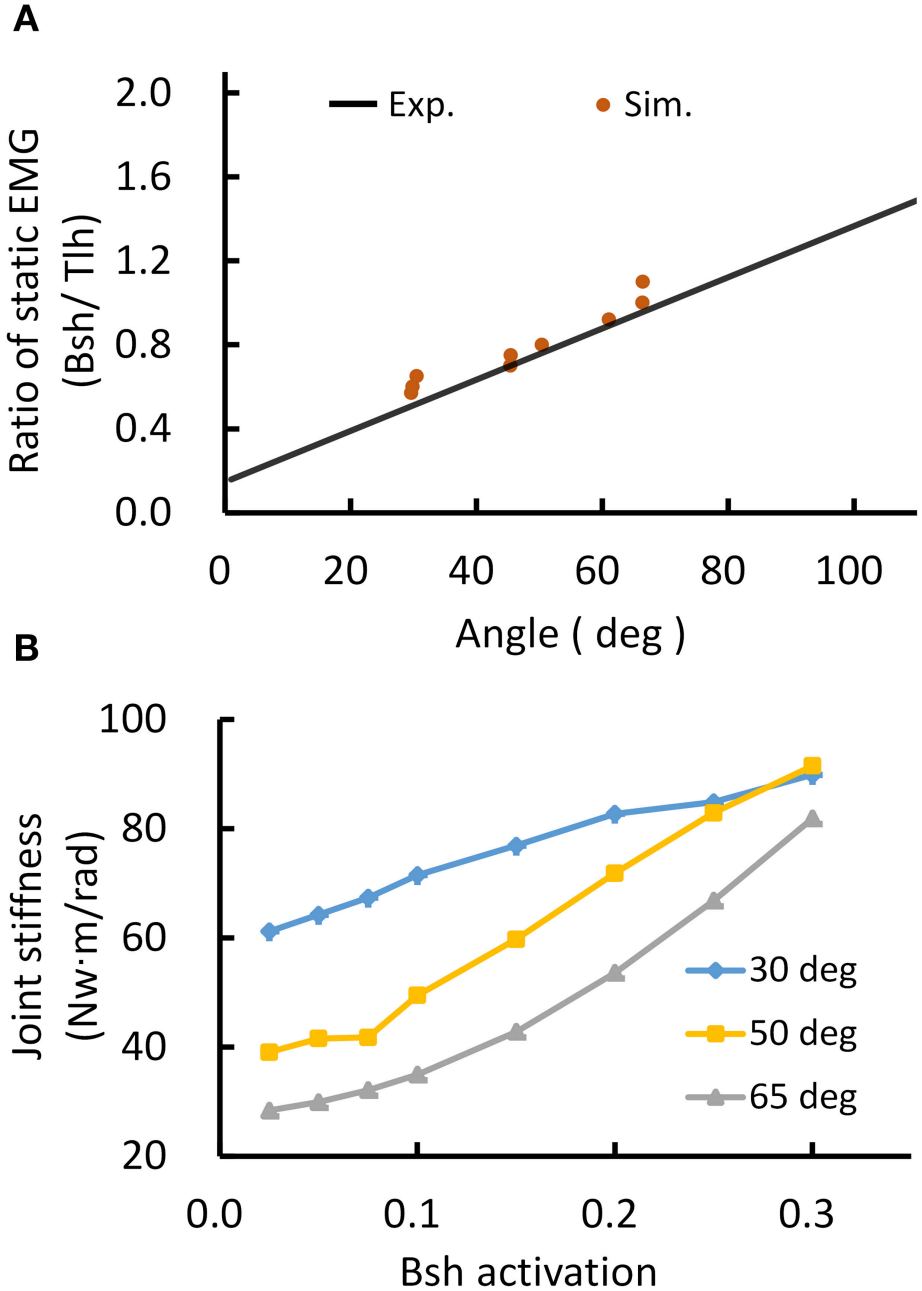
**Simulated posture control. (A)** Muscle activation ratio of simulated postures in comparison with experiment's linear regression line. Different postures were maintained while keeping the ratio of muscle activation of Bsh and Tlh within the range of experimental value. **(B)** Postures maintained with increasing joint stiffness. The elbow angles were maintained at 30°, 50°, and 65° by increasing muscles activation on Bsh, but keeping the ratio of Bsh and Tlh within experimental range, independently controlling joint stiffness was realized while maintaining the same posture.

Thus, the posture module of motor system can control joint stiffness while maintaining the same joint posture. Figure 5 demonstrated such a strategy by increasing the co-activation levels of antagonistic muscles for Bsh and Tlh, while keeping the γ_*s*_ commands at the values corresponding to elbow angles of 30°, 50°, 65° respectively. Figure 5A showed that the simulated posture angles against the experimental linear relation. Since multiple activation levels of muscles can achieve the same elbow posture, joint stiffness can be controlled to desired levels for different motor tasks. We calculated joint stiffness by applying force perturbations on BS and measured the ratio between change of joint torque and angle. Figure 5B showed that joint stiffness increased with the muscle activation, while the same posture was maintained. The results indicate that the central motor system can control joint stiffness and posture independently by tuning the levels of α_*s*_ commands with programmed γ_*s*_ control for postures.

### Simulated movement control

**Figure 6:**
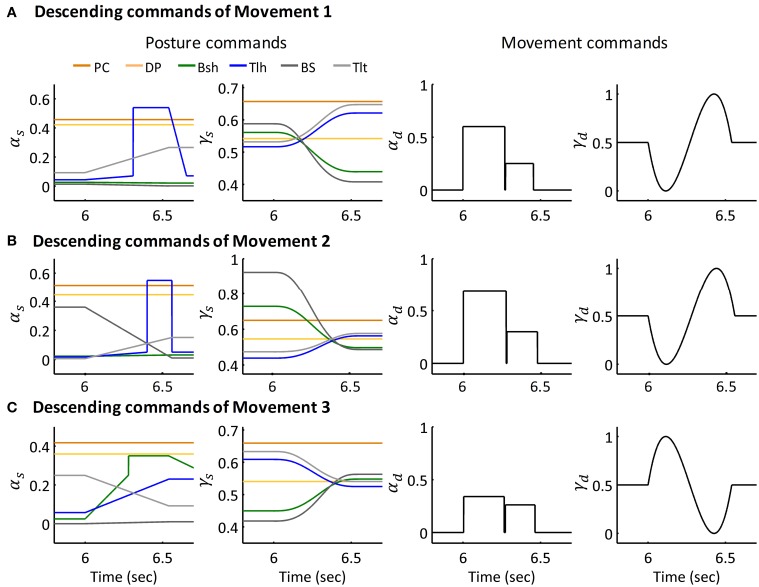
**Descending commands of Simulated Movement 1, 2, and 3 for Subject 1. (A)** Descending commands of Movement 1. The joint was held at initial posture of 63.36° and terminal posture of 29.67° by posture commands, the amplitude and pulse width for AG1 (Tlh) and ANT (Bsh) were 0.60, 0.27 (sec) and 0.25, 0.18 (sec) respectively. **(B,C)** Present the descending commands for movements 2 and 3 respectively. The amplitudes and pulse widths of α_*d*_ for Movement 2 were *amp*_*AG*1_(0.69), *pw*_*AG*1_(0.28(sec)), *amp*_*ANT*_(0.30), *pw*_*ANT*_(0.20(sec)), in Movement 3, they were *amp*_*AG*1_(0.34), *pw*_*AG*1_(0.26(sec)), *amp*_*ANT*_(0.26), *pw*_*ANT*_(0.19 (sec)).

The extension movement from 60° to 30° (Movement 1) was simulated for six subjects individually. An extension movement from 90° to 45° (Movement 2), a flexion movement from 30° to 60° (Movement 3) were simulated for Subject 1. Figures 6A–C depict the descending commands tuned for Movement 1, 2, and 3 respectively. During commands assignment, γ_*s*_ and γ_*d*_ commands were calculated from the MJT of target trajectory (Equations 4 and 5). And the initial and terminal postures were maintained by setting the ratio of α_*s*_ of Bsh and Tlh as experimental values. Thus, movements between postures were simulated with tuning of the pulse height and width of the α_*d*_ command. The amplitudes and pulse widths were adopted from experimental EMG (Equation 6). The third pulse in the α_*s*_ of Tlh acted to help stabilize the elbow after fast movement. The posture commands were also specified for the other four muscles in the model to keep the simulation running properly.

**Figure 7:**
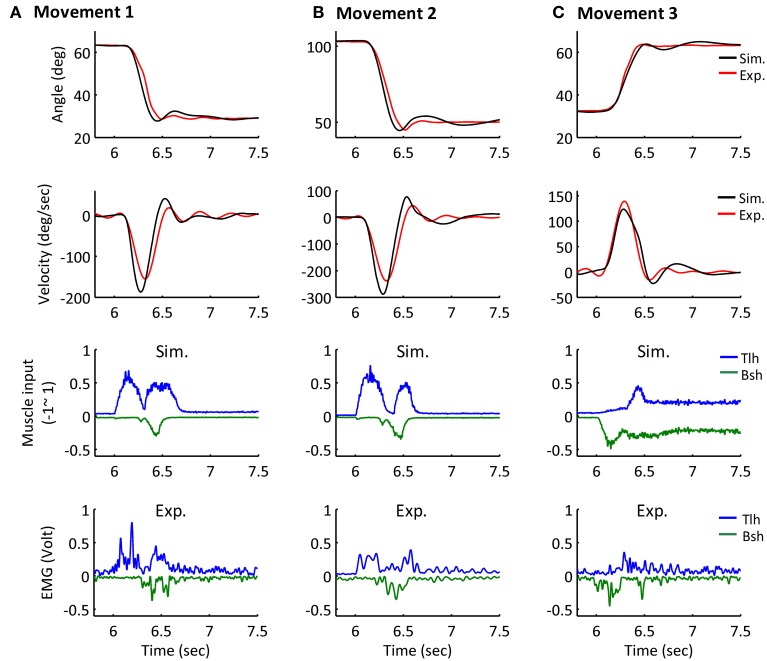
**Simulated Movement 1, 2, and 3, in comparison with experimental data, and they are presented in (A–C) respectively**. The angle and velocity profile were low-passed filtered with the cut off frequency 10 Hz, and the simulated and experimental muscle activation were low-passed filtered with the cut off frequency 50 Hz (Bsh with inverse value). The *Error*_*P*_ of Movement 1, 2, and 3 were 0.19 (deg), −0.04 (deg) and −0.25 (deg), and their *Error*_*M*_ were −3.99 (deg), −5.66 (deg), and −1.44 (deg).

The simulated angle, velocity and muscle activation of Movement 1, 2, and 3 were compared with the experimental movements (Figures 7A–C). The initial and terminal angles could finely match the experimental postures, and the three movements displayed the signature bell-shaped velocity profile, and tri-phasic firing pattern of Bsh and Tlh. Results showed that the elbow could be maintained closely at terminal postures, and the errors during movements were relatively small compared to the range of movements.

Furthermore, all simulated movements matched well to those of experimental trajectories and EMGs in all subjects by tuning the α_*d*_ pulse command. The errors of trajectory fitting for Movement 1 were calculated according to Equation (7) and listed in Table 3. It is demonstrated that the movement module of the central motor system could control movements of a range of angles and velocities by tuning the α_*d*_ pulse command while coordinating posture commands.

**Table 3:** **Fitting Experimental Data of Posture and Movement**.

**Subject**	**Fitted movement**	***Error*_*P*_(*deg*)**	***Error*_*M*_(*deg*)**
S1	Movement 1	0.19	−3.99
S2	Movement 1	0.02	−1.74
S4	Movement 1	0.10	−2.23
S5	Movement 1	0.30	−3.09
S6	Movement 1	0.04	−3.69
S7	Movement 1	0.02	−2.91
Average	Movement 1	0.11	−2.94

*Error_P_ and Error_M_: the mean difference of θ_el_ between simulated and experimental trial (Equation 7), during terminal posture and movement, respectively*.

### Simulation with abnormal afferent and efferent controls

**Figure 8:**
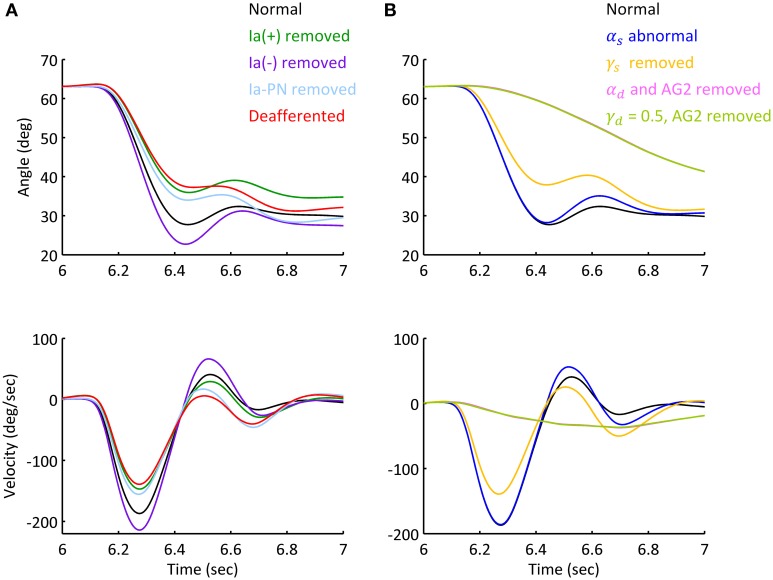
**Simulated movement with abnormal afferent and efferent controls**. The simulations were based on Movement 1 of Subject 1. **(A)** Simulated elbow angle trajectory and velocity under conditions of abnormal afferent feedback. Each feedback gain of Ia afferent was set to zero prior to movement initiation in separated trials. The steady state angle of Ia (+), Ia (−), Ia-PN removed and deafferented conditions were 33.20°, 27.53°, 29.71°, and 31.03°. **(B)** Simulated elbow trajectory and velocity with abnormal static and dynamic efferent commands. The joint terminated at 30.58° under doubled α_*s*_. After γ_*s*_ was removed, the terminal angle was 32.45 (±0.42)°, and the standard variation was 156% of that under normal condition.

Movements under abnormal afferent and efferent controls for Movement 1 were simulated to assess the individual contribution of each descending command to movement and posture. Figure 8 illustrated the various effects for Subject 1. Figure 8A showed that deafferentation after Ia(−) was removed overshot the target of movement with a smaller steady state angle; while removing Ia(+) and Ia-PN undershot the target of movement, note that removing Ia(+) resulted in a larger steady state angle, while Ia-PN had no effect on final posture. When all Ia afferents to αMN were removed in deafferented state, the joint undershot the target of movement, and stabilized gradually to a slightly larger steady state posture.

The results of abnormal efferent commands were shown in Figure 8B. When the ratio of α_*s*_ was doubled, the movement and steady state posture were slightly affected compared to those in normal condition. When γ_*s*_ was removed, the joint undershot the target of movement similar to that of deafferentation. After α_*d*_ was removed or γ_*d*_ kept a constant inhibition on PNs, the elbow couldn't make the fast movement as in normal condition. The slow approach to the target posture was brought about by the action of spinal reflexes. Thus, feedforward control of descending commands (α_*d*_ and γ_*d*_) is absolutely essential for fast movements.

**Figure 9:**
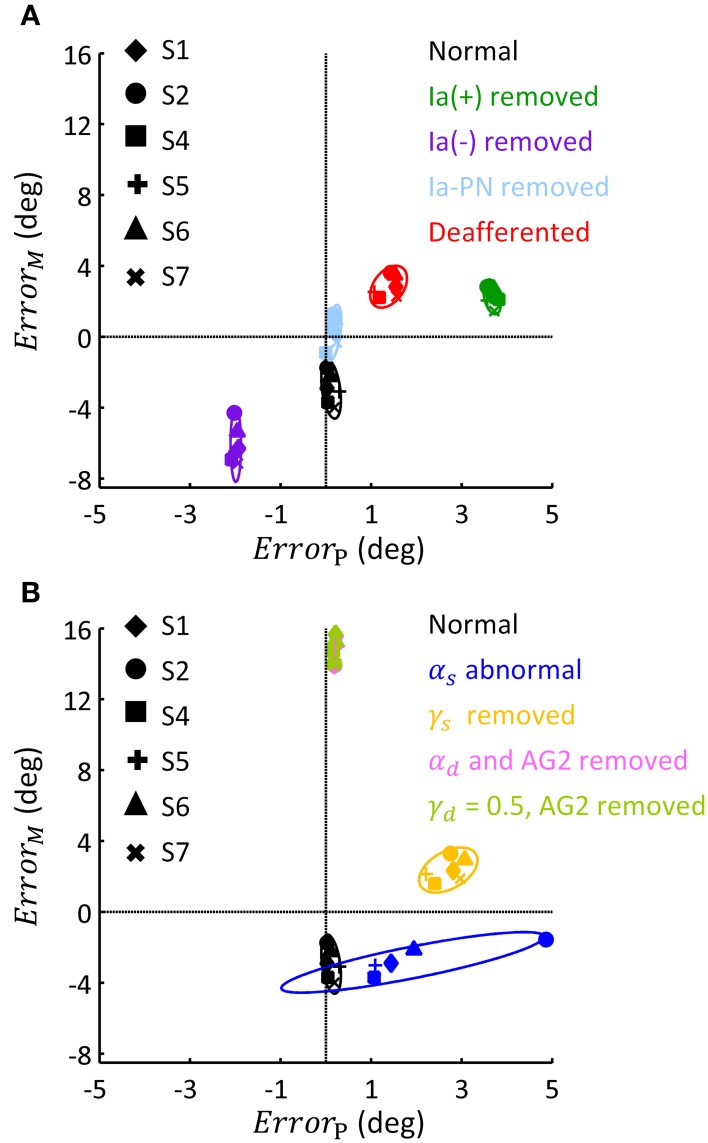
**Phase diagram of the effect of abnormal afferent feedback and efferent commands on posture and movement for six subjects**. All simulations were based on the Movement 1, shown in Table 3. **(A)** The errors of terminal posture and movement comparing with experimental trials, without Ia(+), Ia(−), Ia-PN, and all of them, respectively. *Error*_*P*_ and *Error*_*M*_ are the mean difference of θ_*el*_ between simulated and experimental trial, during terminal posture and movement, respectively. The dashed lines represented no posture or movement error. **(B)** The errors of terminal posture and movement comparing with experimental trials, with abnormal α_*s*_, blocked γ_*s*_, blocked α_*d*_ and constant γ_*d*_, respectively. The average of *Error*_*P*_ and *Error*_*M*_ was 0.21(deg) and 14.75 (deg) for condition of α_*d*_ removed, and 0.20 (deg) and 14.92 (deg) for condition of γ_*d*_ kept constant.

This general pattern of behavior in all subjects is summarized in the phase diagram of errors in Figure 9. As illustrated in Figure 9A, removing Ia(+), Ia(−) and all Ia afferents generally impacted the terminal postures, these results were consistent with the behaviors observed in humans and animals (Polit and Bizzi, 1979; Gentilucci et al., 1994; Gordon et al., 1995). But removing Ia-PN seemed to reduce errors in both movement and posture. Static and dynamic descending commands showed distinct impacts on posture and movement (Figure 9B). The deviated ratios of α_*s*_ for Bsh and Tlh from normal values yielded a wider departure from the target posture, which was different for individual subjects. The absence of γ_*s*_ resulted in an error primarily in posture. The importance of dynamic commands for movement was clearly seen from the large *Error*_*M*_ in Figure 9B. This is consistent with the observation in cats after the PNs innervating the upper extremity muscles were removed (Alstermark et al., 1981; Alstermark and Isa, 2012).

## Discussion

An important issue in motor control has been to differentiate the role of the γ motor system from that of the α motor system (Stein, 1974; Houk and Rymer, 1981; Bizzi et al., 1991). Although increasing evidence revealed α-γ co-activation during movement and posture control (Vallbo, 1971; Taylor et al., 2006; Prochazka and Ellaway, 2012), the dominant effects of the α motor system tended to diminish the function significance of the γ motor system. Using a computational virtual arm (VA) model (He et al., 2013), Lan and He (2012) re-interpreted a set of experiment data from Stein et al. (2004), Cordo et al. (2002), Taylor et al. (2006), and suggested that γ_*s*_ may encode centrally planned information of joint angle and reinforce the planned joint angle though regulating spindle sensitivity. By coupling the VA model with a corticospinal (CS) network of PN in analyzing involuntary oscillatory movements (Hao et al., 2013), we further hypothesized that γ_*d*_ represents centrally planned joint acceleration. In this paper, the combined CS-VA model was used to implicate the necessity of α-γ coordination in movement and posture control. Only the set of α-γ descending commands were adjusted to fit human movement data. Close match of model behaviors to those observed in human experiments as demonstrated in the results (Figure 7, Table 3) established the validity of the model, therefore, providing a neurophysiologically realistic, multi-scale computational model to evaluate the contribution of various components of descending control in sensorimotor functions. In particular, this model will also be valuable to understand sensorimotor dysfunctions (Hao et al., 2013) and to design novel rehabilitation strategies for motor relearning (Zhuang et al., 2015).

A distinct feature of this computational model is the division of sensorimotor control into movement module and posture module by the dynamic and static descending command sets (Bizzi et al., 2008; Diedrichsen and Classen, 2012). Results of this study indicate that it is possible to coordinate the two sets of descending α-γ commands to achieve accurate control of movement dynamics and stable maintenance of final posture. Methods adopted in this study to determine descending commands are novel, in that these descending commands are specified according to proven rules of central planning for kinematics (Hogan, 1984; Flash and Hogan, 1985) and EMG data collected in human subjects performing reach-and-hold tasks. Assumptions are also adopted from previous analytical studies regarding γ_*s*_ encoding of joint angle (Lan and He, 2012), and γ_*d*_ representation of joint acceleration of planned movement trajectory (Taylor et al., 2006; Hao et al., 2013). Results in Figure 5 and Table 2 indicate that α_*s*_ and α_*d*_ commands can be tuned based on EMG signals of human data at steady state and during movement. Figure 5 shows that tuning α_*s*_ based on the linear ratio of antagonistic muscles in Figure 4 is necessary to achieve independent control of joint posture and stiffness, which is an important aspect of regulation of motor functions by the central motor system (Mussa-Ivaldi et al., 1985). For movement control, the α_*d*_ command is integrated with γ_*d*_ command at the PN to distribute properly the activation to flexor or extensor acting at the joint, and its pulse amplitude and width can be tuned according to the speed of movement and duration of EMG bursts. We illustrated that adjusting these descending commands can fit reach-and-hold movements for a range of amplitude and direction in different subjects. The ability of the model to fit experiment movements suggests that the computational model captures the neural mechanism of corticospinal computation, as well as the modular nature of organization and coordination of descending α-γ commands by the central motor control system (Ghez et al., 2007; Scheidt and Ghez, 2007; Scheidt et al., 2011; Poston et al., 2013).

The model predicted the contribution of descending α-γ commands to movement and posture by deefferenting the CS-VA model in simulation. With abnormal α_*s*_ command out of the range of experimental ratio (Figures 8B, 9B), the terminal angle deviated from its targeted position. The large variation shown at the terminal position suggests that accurate γ_*s*_ command is essential for posture maintenance. The tardy movements under abnormal α_*d*_ and γ_*d*_ (Figure 9B) indicated that fast reaching movement must be performed with proper coordination of α_*d*_ and γ_*d*_ commands. This is consistent with the observation that cats were unable to carry out skilled reaching movement without PN (Alstermark et al., 1981; Alstermark and Isa, 2012).

Proprioceptive afferents from muscle spindle are important for motor learning (Jeannerod, 1988; Schmidt and Lee, 2011), but are not found indispensible for control of movements, since deafferentation in human patients and animals did not entirely disable their movement execution. Early study in deafferented patients indicated obvious motor dysfunction only with larger errors in terminal position acquisition (Polit and Bizzi, 1979; Gentilucci et al., 1994; Gordon et al., 1995) and lower joint stiffness during posture maintenance (Bizzi et al., 1984), but changes in movement kinematic and muscle firing pattern were not obvious (Taub et al., 1966, 1975; Vaughan et al., 1970; Rothwell et al., 1982; Bizzi et al., 1984; Gordon et al., 1995). This implied that proprioceptive afferent played an important role in posture maintenance and contributed to fine control of movement.

This functional role of proprioceptive afferents is reiterated by deafferenting the CS-VA model (Figures 8A, 9A). It was shown that, when Ia(+) and Ia-PN was removed respectively, the movement slowed down, and the terminal posture shifted, and after Ia(−) was removed, the movement speeded up, and the terminal posture targeted at a lower angle. This confirmed the positive feedback of Ia(+) and Ia-PN, and inhibition role of Ia(−). However, despite the difference of peak velocity, these movements presented similar bell-shaped velocity profile, only settled at different terminal angles. Deafferented simulation showed slowed movement and increased errors in terminal angle. This was similar to the behaviors observed in deafferented primates (Polit and Bizzi, 1979), which showed that the deafferented primate after intensive training was able to control pointing movement with normal like kinematics and muscle activation, but inaccurate positions.

## Conclusion

A corticospinal computational model based on the modular organization of movement and posture was validated in this study by fitting the model to experimental human data. Analysis of simulated movement and posture with intact and altered model structures demonstrated that it is necessary to coordinate the set of α-γ descending commands in order to achieve effective control of accurate movement dynamics and stable postures. Results suggest that the central commands of posture module are mediated via a mono-synaptic corticospinal pathway, while those of movement module are transmitted to spinal motoneruons through a multi-synaptic corticospinal pathway involving the propriospinal neurons (PN). The PN network plays the pivotal role to integrate the α_*d*_ and γ_*d*_ commands for movement generation. The model is able to capture many essential aspects of motor behaviors, such as independent regulation of joint angle and stiffness and the signature temporal pattern of EMGs, by simply tuning the α_*s*_ and α_*d*_ commands. This study suggests a plausible neural computational mechanism for the central motor system to control movement and posture. The model will be useful as a complementary tool to understand neural control of movements, as well as a valuable platform to aid design of novel rehabilitation strategy for motor disabilities.

## Author contributions

SL performed model simulation, analyzed experimental data, prepared figures and tables and drafted the manuscript; XH and MH contributed to set up the computational model; JM and CZ contributed in the analysis of human experiment data. CZ also carried out part of simulation work. CN offered intellectual suggestions and edited the manuscript. NL conceived the computational approach, designed the human subject experiment, proposed analytical method and edited the final version of the manuscript.

### Conflict of interest statement

The Chief Editor Prof Wu declares that, despite having sharing affiliation with the Reviewer Dr. Wang, the review process was handled objectively and no conflict of interest exists. The authors declare that the research was conducted in the absence of any commercial or financial relationships that could be construed as a potential conflict of interest.

## Abbreviations

α, γ: Alpha, gamma motor nerves and neurons
PN: Propriospinal neuron
EMG: Electromyography
GTO: Golgi Ten Organ
CS-VA: Corticospinal virtual arm
VA: Virtual arm
α_*s*_, γ_*s*_: Static alpha, gamma command
α_*d*_, γ_*d*_: Dynamic alpha, gamma command
αMN, γMN: Alpha, gamma motoneuron
*U*_*m*_: Muscle input in the CS-VA model
*U*_*s*_: Spindle input in the CS-VA model
*F*_*m*_: Muscle force
*L*_*ce*_: Muscle fascicle length
*L*_*mt*_: Musculo-tendon length
Ia: Primaryafferent from spindle
II: Secondary afferent from spindle
Ib: Afferent from GTO
PC: Pectoralis Major Clavicle
DP: Deltoid Posterior
BS: Brachialis
Tlt: Triceps Lateral
Bsh: Biceps Short Head
Tlh: Triceps Long Head
θ_*el*_: Elbow angle
θ_*sh*_: Shoulder angle
γ-PN: Dynamic gamma inhibition on PN
Ia(−): Ia reciprocal inhibition on αMN
Ia-PN: Ia afferent gain on PN
Ia(+): Stretch reflex
Ib(−): Ib inhibition from GTO
RC: Renshaw Cell
*Error*_*P*_*, Error*_*M*_: mean difference between simulation and experiment of terminal posture, movement
*V*_*peak*_: Peak velocity
3SD: Three times of standard deviation of static EMG
AG1: First agonistic muscle burst phase
AG2: Second agonistic muscle burst phase
ANT: Antagonistic muscle burst phase
RS: Ratio of static EMG (Bsh/Tlh)
RP: Ratio of peak EMG (ANT/AG1)
*amp*_*AG*1_*amp*_*ANT*_: Amplitude of AG1 and ANT phase on α_*d*_
*pw*_*AG*1,_*pw*_*ANT*_: Pulse width of AG1 and ANT phase on α_*d*_
MJT: Minimum jerk trajectory.
